# Influence of Ultraviolet Radiation on Mechanical Properties of a Photoinitiator Compounded High Vinyl Styrene–Butadiene–Styrene Block Copolymer

**DOI:** 10.3390/polym13081287

**Published:** 2021-04-15

**Authors:** Sanjoy Datta, Radek Stocek, Kinsuk Naskar

**Affiliations:** 1 Centre of Polymer Systems, Tomas Bata University in Zlín, tr. Tomase Bati 5678, 760 01 Zlin, Czech Republic; stocek@utb.cz; 2 Indian Institute of Technology Kharagpur, Kharagpur 721302, West Bengal, India; knaskar@rtc.iitkgp.ac.in

**Keywords:** ultraviolet radiation, thermoplastic elastomer, high vinyl S-B-S, photoinitiator, mechanical properties

## Abstract

Ultraviolet curing of elastomers is a special curing technique that has gained importance over the conventional chemical crosslinking method, because the former process is faster, and thus, time-saving. Usually, a suitable photoinitiator is required to initiate the process. Ultraviolet radiation of required frequency and intensity excites the photoinitiator which abstracts labile hydrogen atoms from the polymer with the generation of free radicals. These radicals result in crosslinking of elastomers via radical–radical coupling. In the process, some photodegradation may also take place. In the present work, a high vinyl (~50%) styrene–butadiene–styrene (SBS) block copolymer which is a thermoplastic elastomer was used as the base polymer. An attempt was made to see the effect of ultraviolet radiation on the mechanical properties of the block copolymer. The process variables were time of exposure and photoinitiator concentration. Mechanical properties like tensile strength, elongation at break, modulus at different elongations and hardness of the irradiated samples were studied and compared with those of unirradiated ones. In this S-B-S block copolymer, a relatively low exposure time and low photoinitiator concentration were effective in obtaining optimized mechanical properties. Infrared spectroscopy, contact angle and scanning electron microscopy were used to characterize the results obtained from mechanical measurements.

## 1. Introduction

Light-induced polymerization is one of the most effective methods to generate three-dimensional polymer networks, because of the high initiation rates reached under intense illumination [1,2,3]. In most UV-curing applications, a solvent-free liquid resin is converted quasi-instantly into a highly crosslinked polymer, selectively in the exposed areas, to produce protective coatings, quick-setting adhesives or high-resolution relief images. The photochemical process has been widely used to crosslink solid polymers with polymerisable functional groups on their backbones [4], e.g., cinnamates [5], epoxides [6,7] and acrylates [8]. A distinct advantage of photoinitiation is to afford precise control of the chemical process. The crosslinking reaction is instantaneous, and starts immediately with the impingement of light of suitable frequency, and it can be stopped by switching off the UV lamp. The rate of reaction of course varies as a function of UV beam intensity.

UV-crosslinking of epoxy [9,10,11] or acrylate [12,13] functionalized natural rubber in presence of suitable photoinitiators have been studied successfully, but natural rubber alone does not show any reaction under such condition because of the low reactivity of the amylene double bond. In styrene–butadiene–styrene (SBS), it had been recently reported that the vinyl-functionalized mid-block can be readily photocrosslinked by UV irradiation at ambient temperature in the presence of a suitable photoinitiator. The pendent vinyl double bonds are known to be more reactive than the in-chain butene double bonds of the polybutadiene segments [14]. They are thermoplastic elastomers in nature and exhibit mouldability like thermoplastics at elevated temperatures and the functional performance of elastomer at ambient temperatures. Literature survey shows that very limited work related to UV-curing of SBS copolymer is openly published because of the commercial sensitivity. In particular, photocuring of SBS polymers was extensively studied by Decker et al. [15,16,17].

The authors have successfully presented a comprehensive investigation using statistical design of experiments (DOE), using design expert software pertaining to response surface methodology (RSM) to identify the influential effects of the process variables on the final physical properties of UV-photocured SBS block copolymer [18]. The aim of that work was to mathematically understand the effects of process parameters (time and distance) as a function of photoinitiator (PI) concentration and molecular characteristics (vinyl content) on the physico-mechanical properties of UV-cured SBS block copolymer. In that study, it was found that relatively lower exposure time at lower photoinitiator concentration with a closer distance from the UV source on a higher vinyl content polymer produced the optimum condition for the overall balance of mechanical properties.

Based on the results obtained in the previous work, this study was framed. The main objective of the present work was to study the effect of UV radiation on the mechanical properties of the polymer using 4,4′ dihydroxybenzophenone as the photoinitiator at various concentrations, each of the batches subjected to two different exposure time of 15 s and 30 s and to correlate the results obtained with the previous paper based on the optimisation of photoinitiator concentration and time. Further, the authors were interested to see improvement in the mechanical properties with the use of the new photoinitiator over the previously used benzophenone serving the same purpose. The results obtained were supported through attenuated total refraction (ATR) Fourier transform infrared (FT-IR) spectroscopy, contact angle and microscopic characterisation of the batches used for the study.

## 2. Materials and Methods

### 2.1. Materials

**Figure 1:**
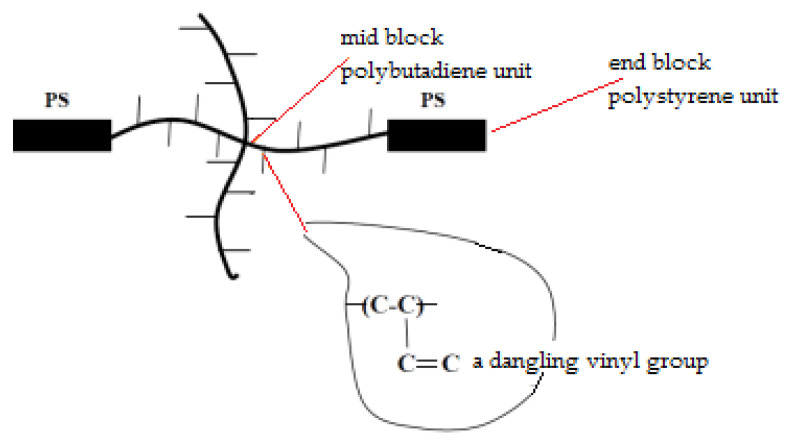
A two-dimensional structure of high vinyl styrene–butadiene–styrene (SBS) block copolymer.

Styrene–butadiene–styrene (S-B-S) block copolymer Kraton DKX222 was obtained from Kraton Polymers, Belgium. It contains 18 weight percent bound styrene and 82 weight percent bound butadiene. The microstructure of the polybutadiene midblocks is about 50% 1,4(trans, cis) and 50% 1,2(vinyl) insertion in a random sequence. It has a density of 910 kg/m^3^ and weight average molecular weight <Mw> = 71,000 [19]. The structure of the polymer is shown in Figure 1 [14]. It is also seen from Figure 1 that there are dangling groups in the polymer main chains and these groups are due to 1,2(vinyl) insertion during polymerisation. In this figure, PS represents the end block polystyrene units while the midblock polybutadiene units (represented by the curved lines are seen to house the dangling vinyl units shown as smaller protruding straight lines.

### 2.2. Preparation of the Batches

Batches of the high vinyl SBS copolymer varying in the PI concentration were prepared in a Haake Rheomix OS 600 (Thermo Fisher Scientific GmbH, Karlsruhe, Germany), with a mixer chamber volume of 85 cm^3^. Each batch size was around 55 g and the mixer temperature was kept between 90–100 °C. A constant rotor (cam type) speed of 65 rpm was applied. After 2 min of homogenization of the polymer mass, appropriate amount of the PI was added and the mixing was completed in 6 min. Immediately after each mixing, the composition was removed from the mixer, and while still in hot condition, passed once through a cold two-roll mill to achieve a sheet of about 2 mm thickness. The sheet was cut and pressed (2 mm) in a compression molding machine (George Moore press, UK), at 120 °C, for 5 min and 3.94 × 104 kg/m^2^ ram diameter pressure. While molding, TeflonVR sheets were placed between the sheet and the hot plates. The sheet was then cooled to room temperature by circulating cold water through the press plates.

The sheets with a thickness of around 2 mm were subjected to ultraviolet treatment of appropriate doses as shown in Table 1.

UV radiation was carried out using an Ultraviolet Medium Pressure Quartz Lamp with a wavelength of about 250–350 nm (Advanced Curing System, Bangalore, India). Samples were exposed to the radiation under 1800W mercury lamp, in the presence of air, at a defined time and packing height. The maximum light intensity at the sample position was measured by radiometry (IL-390 light bug) to be 600 mW cm^−2^. The samples were designated as k_UV_, k to represent the Kraton polymer used and _UV_ signifying ultraviolet radiation treatment. This was followed by the numbers 0.2, 0.4, 0.06, 0.8, 1.0 and 1.5 corresponding to the PI concentration in phr. Finally, the numbers 15 and 30 showed the time of exposure of the samples to UV radiation.

### 2.3. Testing Programs

#### 2.3.1. Mechanical Characterization

Tensile tests on the treated and untreated samples were performed according to ASTM D 412 on dumbbell-shaped specimens (Type 2) using a Hounsfield tensile testing machine H10KS (Germany) at a constant crosshead speed of 500 mm/min.

“Shore A” hardness of the samples was measured using a Durometer type A, as per ASTM D 2240.

#### 2.3.2. Spectroscopic Characterization

Fourier Transform Infrared (FT-IR) Spectroscopic Analysis of the unirradiated and irradiated samples were done in attenuated total reflection (ATR)-FTIR spectra in the range of 4000 to 650 cm^−1^ using an infrared spectrophotometer (Nicolet Nexus, Madison, WI, USA). The spectra were obtained at a resolution of 4 cm^−1^ using a zinc selenide crystal. The data obtained from the spectrometer were then fed in an algorithm of baseline creation and subsequent subtraction [20,21,22,23] to quantify the disappearance of the vinyl pendant groups showing peak at 909 cm^−1^ [24], which actively participated in the photocrosslinking process. The quantification was done against normalized peak of polybutadiene unit at 965 cm^−1^ [24] of the SBS block copolymer.

#### 2.3.3. Calculation of Surface Energy by Contact Angle Method

The contact angles of different liquids on UV-irradiated samples were obtained using a Ramé Hart contact angle meter. Before the UV treatment, the samples were compression moulded within Mylar (polyester) films to keep them dust-free. Only during the brief time of exposure of the samples to UV, the films were temporarily removed. After the UV treatment, the samples were again covered on both sides with the Mylar films. During the contact angle measurement, the surfaces of the samples were exposed by removing the covers. All investigations were carried out using polymer plates which were cut out from the moulded and UV-crosslinked sheets to obtain dimensions of 10 mm × 10 mm × 2 mm. This produced an almost perfectly flat surface for contact angle measurements.

The sessile drop method employing 4 μL drops of different probe liquids was applied for the contact angle measurements. The liquids used for the contact angle measurements were bi-distilled water, formamide and diiodomethane. Each contact angle value quoted was the mean of at least three measurements with a maximum error of ±1°. All investigations were performed in air at 25 ± 1 °C. the experiments were carried out up to exactly 5 min. Surface energies of the UV-crosslinked samples were calculated equating the measured contact angle (θ) to the free surface energy using the Owens and Wendt equation Equation (1) [25]

(1) $$\cos\theta =-1+\frac{2{\left(\gamma_{s}^{d}\cdot \gamma_{l}^{d}\right)}^{1/2}}{\gamma_{l}}+\frac{2{\left(\gamma_{s}^{p}\cdot \gamma_{l}^{p}\right)}^{1/2}}{\gamma_{l}}$$

where *γ^d^* and *γ^p^* are the dispersive and the polar components respectively of the free surface energy of solid and liquid, (*s* = solid and *l* = liquid). To find the contact angle, Rame Hart goniometer (Rame Hart Instrument Co, Succasunna, NJ, USA) was used. Bidistilled water, formamide and diiodomethane were selected as the probe liquids. The surface parameters of these probe liquids were taken from the literature for calculating contact angle (θ) [26,27].and are shown in Table 2.

#### 2.3.4. Morphological Studies in Raame Hart Camera

To understand the nature of dispersion of UV photoinitiator within the matrix of the variously compounded high vinyl S-B-S block copolymer, visible light was passed through selected samples and the anterio-postirior photographs were captured in a camera attached with the Ramé Hart contact angle equipment. The images were magnified enough to capture the dispersion. However, the camera did not have the provision to register the value of magnification.

#### 2.3.5. Scanning Electron Microscopic Studies

To examine the surface morphology, scanning electron microscopic (SEM) studies were performed on gold-coated samples using a scanning electron microscope JSM 5800 (JEOL, Tokyo, Japan) at 10 kV at a magnification of 5k.

#### 2.3.6. Crosslink Density Calculation

Crosslink densities were measured using the modified Flory Rehner equation by the equilibrium solvent swelling method. In this case, cyclohexane was chosen as the equilibrium solvent due to its solubility parameter of 8.18 (cal/cm^3^)^1/2^ which is close to that of butadiene units of S-B-S). Initial weight, swollen weight and de-swollen or dried weight were measured and substituted in Equation (2) which is as follows:

(2) $$\nu =-\frac{1}{v_{s}}\cdot \frac{\ln(1-v_{r})+v_{r}+\chi {\left(v_{r}\right)}^{2}}{{\left(v_{r}\right)}^{1/3}+0.5v_{r}}(mol\cdot {ml}^{-1})$$

where:

ν → = number of moles of effectively elastic chains per unit volume of the polymer [mol/mL] (Overall Crosslink Density),
Vs → = molar volume of the solvent (here cyclohexane) used [cm^3^/mol],
χ → = polymer-swelling agent interaction parameter (here, 0.3) (Barton 1985) or Flory–Huggin’s parameter,
Vr → = volume fraction of the polymer in the swollen network, expressed as Vr = 1/(Ar + 1),
Ar → = is the ratio of the volume of absorbed solvent (cyclohexane) to that of the polymer after swelling (Flory and Rehner 1943; Naskar 2004).

## 3. Results and Discussion

### 3.1. Mechanical

The results obtained for the unirradiated as well as the samples irradiated at 15 s and 30 s at various concentrations of the photoinitiator are presented in Table 3.

**Figure 2:**
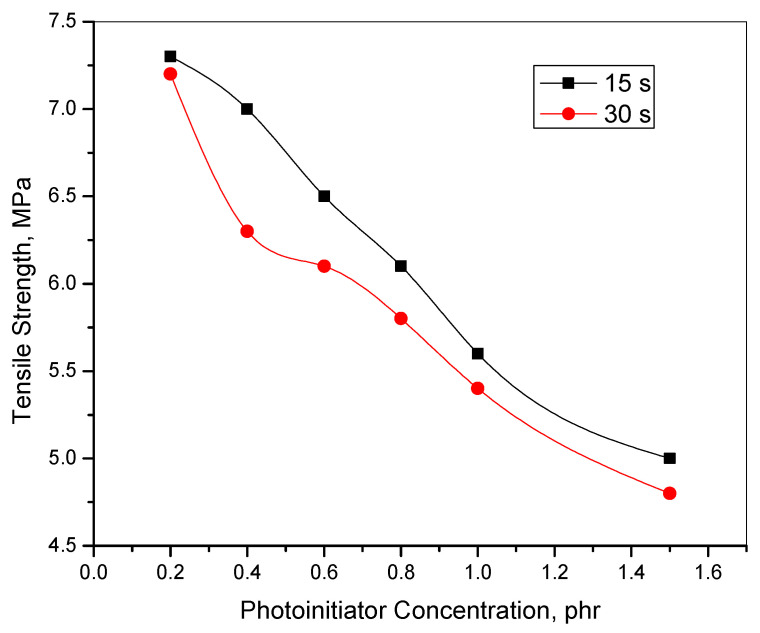
Tensile strength as a function of photoinitiator concentration.

For the UV-irradiated samples, the tensile strength showed a decreasing trend with an increase in photoinitiator concentration with the 15 s crosslinked samples showing marginal higher values at equivalent photoinitiator concentration over the 30 s crosslinked ones. The maximum tensile strength of 7.3 MPa at photoinitiator concentration of 0.2 phr and irradiation time of 15 s was a clear indication of improvement over the unirradiated control sample which had a tensile strength value of 5.3 MPa. The 30 s exposed sample at the same photoinitiator concentration showed a tensile strength of 7.2 MPa from where it may be inferred that an additional exposure time of 15 s, which consumed more energy did not produce any improvement in tensile strength. The results obtained are better visualized in Figure 2.

**Scheme 1:**
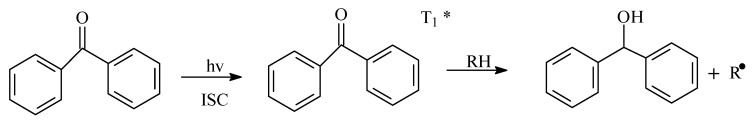
Plausible reaction scheme of 4,4′dihydroxybenzophenone under UV irradiation.

Though all the photoinitiator compounded samples got crosslinked with the generation of free radical sites on the polymer in accordance with Scheme 1 [28], yet with an increase in the photoinitiator concentration the probability of crack initiation in the polymer matrix and subsequent crack propagation was perhaps the most plausible explanation for the reduction in tensile strength.

**Figure 3:**
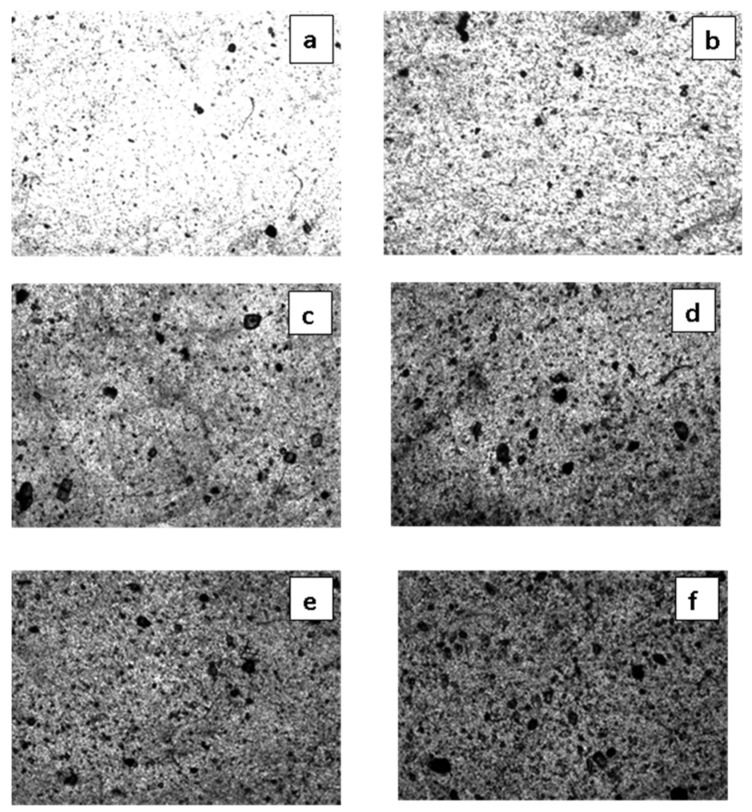
Antirio-posterior photographs of UV-cured samples at (**a**) 0.2, (**b**) 0.4, (**c**) 0.6, (**d**) 0.8 (**e**) 1.0 and (**f**) 1.5 phr of the antioxidant (magnified but not quantified).

Arguably though the surface consumed up all the photoinitiator as will be discussed in the subsequent part of the results and discussion section, yet there was a large excess of un-reacted photoinitiatior in the bulk, acting as an impurity. This excess amount was further supported through photographs captured in a Ramĕ–Hart camera attached with the contact angle goniometer which is presented in Figure 3. These were photographs taken in the antirio-postirior direction with visible light passing through the polymer.

The photographs captured the presence of aggregates of the photoinitiator embedded within the polymer matrix. They clearly proved that with an increase in the photoinitiator concentration, the average size as well as the population of the photoinitiator both increased. The photographs show a gradual increase in darkening shades with an increase in photoinitiator concentration, which conclusively proved the assumption of residual photoinitiator in the bulk.

**Figure 4:**
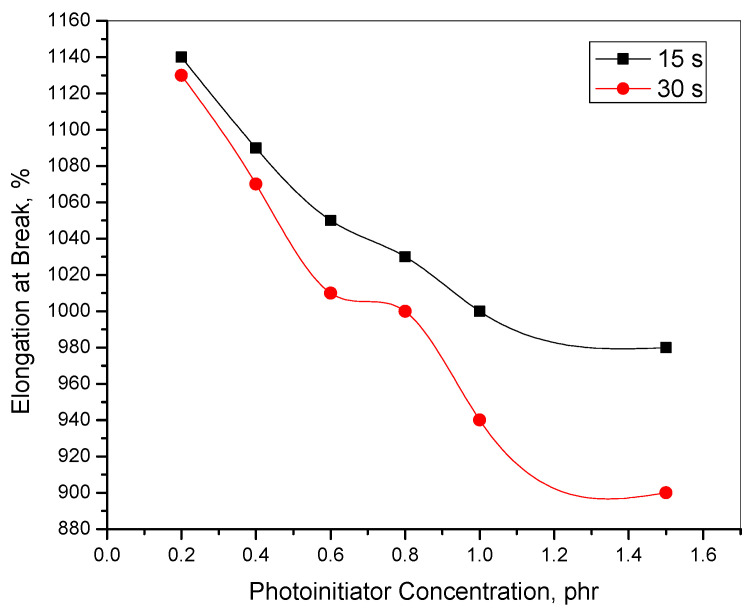
Elongation at break as a function of photoinitiator concentration.

The elongation at break also showed a decreasing trend as a function of the photoinitiator concentration as is evident from Figure 4.

**Figure 5:**
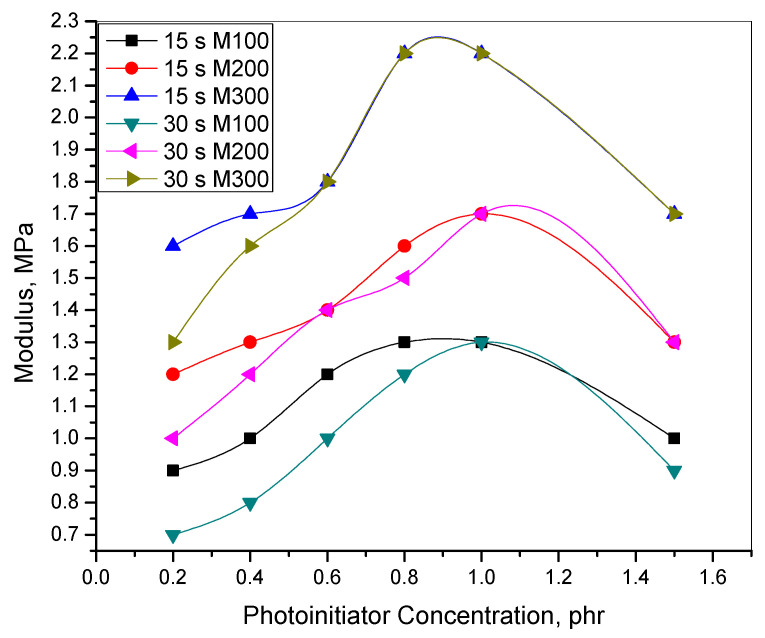
Modulus at 100, 200 and 300% elongations as a function of photoinitiator concentration.

It was reasoned out that the elongation at break was the determining parameter for the tensile strength. Additionally, from Table 3, better understood through Figure 5, it is seen that the M100, M200 and M300 for both 15 and 30 s UV-irradiated samples increased almost linearly from 0.2 to 1.0 phr of the photoinitiator and then decreased at the highest concentration of 1.5 phr.

Here also, it was supposed that at very high photoinitiator concentration, the polymer housed many big aggregates of the unreacted photoinitior in the bulk, which served as potential areas of weaknesses to decrease the magnitude of modulus through the phenomenon of multipoint crack initiation and subsequent crack propagation.

Usually for lowly crosslinked thermoplastic elastomers, as is the case in the present study and as will be shown through the crosslink density calculation, the modulus even at reasonably high elongations increases as a function of crosslink density while the tensile strength increases to a maximum and then decreases. This is because modulus is a function of crosslink density only, while tensile strength depends simultaneously on the crosslink density as well as the amount of energy that can be dissipated from the polymer matrix.

**Figure 6:**
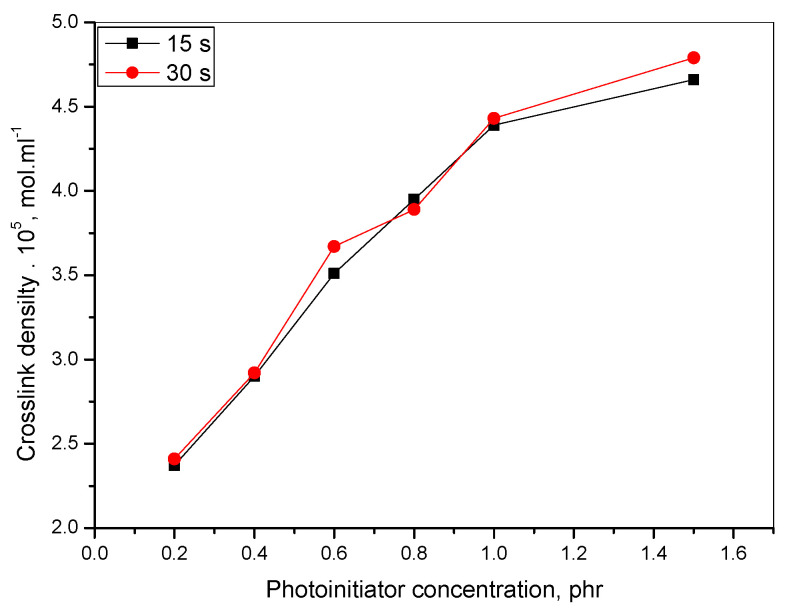
Crosslink density as a function of photoinitiator concentration at 15 and 30 s exposure time.

However, in the present study, it was observed that the crosslink density continuously increased as a function of the photoinitiator concentration, though at a little lesser rate from 1.0 to 1.5, as is shown in Figure 6, but the modulus at any of the three defined elongations decreased from 1.0 to 1.5 phr of the photoinitiator concentration.

In general, crosslink levels should be high enough to prevent failure by viscous flow but low enough to avoid brittle failure. However, in this study, the crosslink density was in the low order of 10^−5^, which could not have enhanced the brittle failure of the polymer. Still, it was observed that the modulus after an initial steady increase finally decreased. The explanation has already been given in terms of crack initiation and propagation.

For elongation at break as well as modulus, the 15 and 30 s crosslinked samples showed comparable results as is evident from Table 3, with the maximum modulus obtained at 1 phr of photoinitiator for both. The modulus values produced marked improvement over the control sample as can be observed from the table.

Hardness measured on the surfaces of the irradiated samples ranging from 50–53 “Shore A” also reflected an increase over the control sample where the hardness was only 41. In general, an increase in hardness is accompanied by an increase in modulus values. However, as was argued earlier, the modulus at the highest photoinitiator concentration decreased as a result of the role played by the residual aggregates of photoinitiator in the bulk.

Along with the postulate of aggregate size playing a role in determining the mechanical properties, another radical explanation may be given to understand the trends observed.

Generally, when irradiated with UV light, benzophenone and substituted benzophenones absorb energy and are excited to singlet state which is not stable. So, they rapidly relax to the more stable triplet state by intersystem crossing (ISC). Studies have found that the excited triplet states are efficient hydrogen abstracting species as shown in Scheme 1. These in turn lead to the formation of polymeric free radicals by absorbing hydrogen from the liable sites [28]. According to Scheme 1, it can be said that during photochemical reaction involving benzophenone type photoinitiator, the total number of macroradical sites depends on the concentration of the photoinitiator.

Thus, the negative shift in the physical properties as a function of photoinitiator concentration as was found in the results of the experiments can be reasoned out by the fact that an increase in photoinitiator concentration has two possible effects. On one hand, it accelerates the crosslinking reaction by the formation of more reactive species. On the other hand, it steepens the cure depth gradient, especially for thick samples, called as “inner shield effect” [17,29]. Hence it was ascertained that as the photoinitiator concentration increased from 0.2 to 1.5 phr, a compromise between effective and insufficient crosslinking in the inner or middle layers of the samples took place and consequently the physical properties were affected in a negative manner. This was true for tensile strength for all the concentrations and correct for modulus above a concentration of 1.0 phr. However, as discussed earlier, this was the second effect, the first one attributed to the aggregate residues of the unreacted photoinitiator in the bulk again due to the inner shielding effect. However, insufficient did not mean that the crosslink density would decrease after a certain concentration of the used photoinitiator.

**Figure 7:**
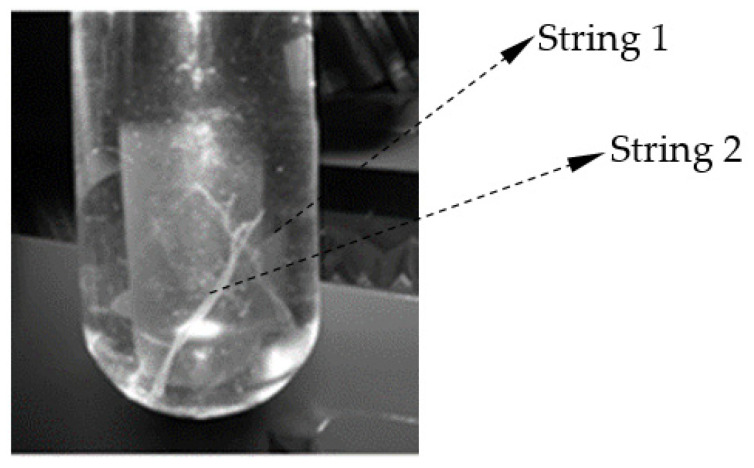
Photograph of UV-crosslinked polymer showing the generation of two strings in cyclohehane after a period of 48 h from the initial time of immersion.

This phenomenon of insufficient crosslinking in the bulk was conclusively proved by carrying out some interesting sol–gel experiments. Cylohexane is a suitable solvent for the SBS block copolymer and when uncrosslinked, the polymer mass formed a monophasic solution in it. However, it was found that each UV-irradiated sample in the presence of the photoinitiator generated two strings after immersing the samples in cyclohexane for a period of 48 h at ambient temperature. The photograph of one such swollen sample at equilibrium with cyclohexane showing the generated strings are shown in Figure 7.

Due to the inner shielding effect, the inside of each of the samples did not get crosslinked and was thus dissolved in the solvent. The two outer surfaces that got crosslinked were rendered insoluble and naturally in their highest entropic condition were manifested as strings. Since the test specimens for the sol–gel experiments were scissor cut from the original UV-irradiated samples, thus they were necessarily cuboidal in shapes, with a length, a breadth (dependent on the cutting) and a thickness of about 2 mm (original moulded thickness before exposure to UV radiation), the strings which were generated after 48 h immersion in cyclohexane were actually very thin UV-crosslinked elements (much thinner than 2 mm), but with the same lengths and breadths of the specimens used before immersion.

Based on this assumption, a very simple but an innovative method was adopted to find the thickness of cure of such a crosslinked system. The relation m = ρ/v, was used for the purpose, where ρ was the density of the compounded and crosslinked sample, m the mass and v the volume all expressed in appropriate units. Knowing the density to be 0.91 g cm^−3^ for the uncompounded and uncrosslinked polymer and assuming that the density did not vary considerably after the addition of a small amount of the photoinitiator in the range of 0.2 to 1.5 phr, it was further assumed, for all practical calculations that the densities of the crosslinked samples were also 0.91 g cm^−3^.

After the formation of the strings (actually each was a cuboidal volume with a definite length, breadth and thickness) and subsequent drying, it was found that the masses of the two strings for each of the photoinitiator concentrations were almost the same. This proved that both the sides exposed to the same time of exposure to UV radiation got crosslinked to the same extent. The volume of a string was obtained by dividing the mass by the density. Since the length and the breadth of the string were assumed to be the same as that of the sample before it was immersed in the solvent, the area of the string remained the same. Dividing the calculated volume by this area provided the thickness of crosslinking of the string.

This was then a direct means to find the thickness of crosslinking without going for any other instrumental methods. For the samples under investigation, the thickness of crosslinking was about 0.30 μm (each side) at photoinitiator concentration of 0.2 phr and 0.22 μm at 1.5 phr. There was no reportable difference between the thickness of crosslinking due to a variation in the exposure time.

**Scheme 2:**
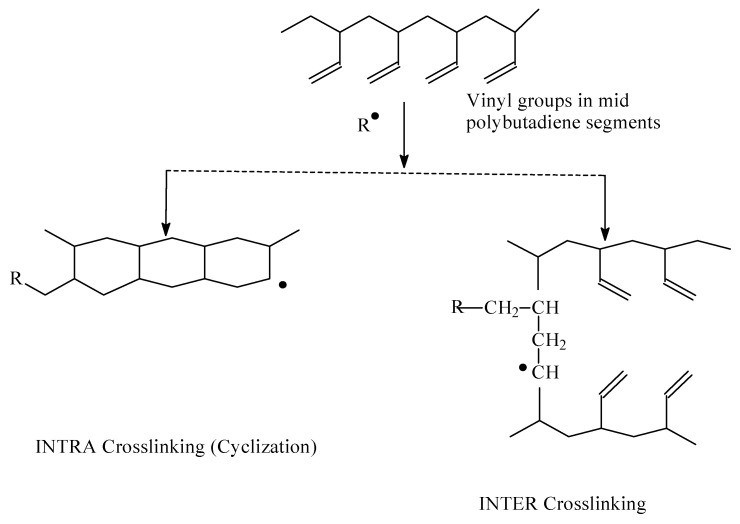
Plausible reaction scheme of vinyl functionality in the butadiene segments.

The vinyl double bond in the SBS block copolymer is more reactive than the in-chain butene double bonds. However, a close look in the vinyl chemistry shows that, in addition to the intermolecular crosslinking, an intra-molecular cyclization or cyclopolymerization process may take place in the vinyl bonds located on the same polybutadiene chains [15]. The latter reaction leads to the formation of hard brittle clusters or domains, which in turn act as stress concentration points to reduce the mechanical properties. Hence, the ultimate physical properties, along with what is discussed so far were again a compromise between the inter- and intra-molecular crosslinking for the high vinyl-SBS samples (Scheme 2) [15]. In addition to that, it may also be reasoned out that a significant rise in the temperature took place due to an exothermic crosslinking reaction. The resulting increase in molecular mobility would favour more crosslinking and hence the formation of a tighter network structure [15].

The rise in temperature, though not monitored in the present study can yet be appreciated according to some researches on some other polymers under the broad head of thermal and thermomechanical properties of block copolymer [30,31].

From what has been discussed so far, it may be inferred that the polymer was productive to UV radiation in the range of 250–350 nm in the presence of 4,4′ dihydroxybenzophenone as the photoinitiator. Although the mechanical properties were very close, it was s the 15 s irradiated samples that showed marginal better tensile properties over the 30 s irradiated ones.

If the overall mechanical properties were improved in a 2 mm thick sample where inner shielding effect prohibited the bulk of the polymer to get crosslinked, then definitely the properties would have been much better if the experiments were carried out with thin films of dimensions in the order of μm.

In fact, the present study with a 2 mm thick sample, compounded by melt mixing process was advantageously used to understand both, what happened under such a condition, and also to predict what may happen if the solvent casting is chosen to get a thin film of the polymer in which the photoinitiator will be more homogeneously distributed with a uniform crosslinking of virtual no inner shielding effect due to the thin film.

### 3.2. Infrared Spectroscopic Studies

**Figure 8:**
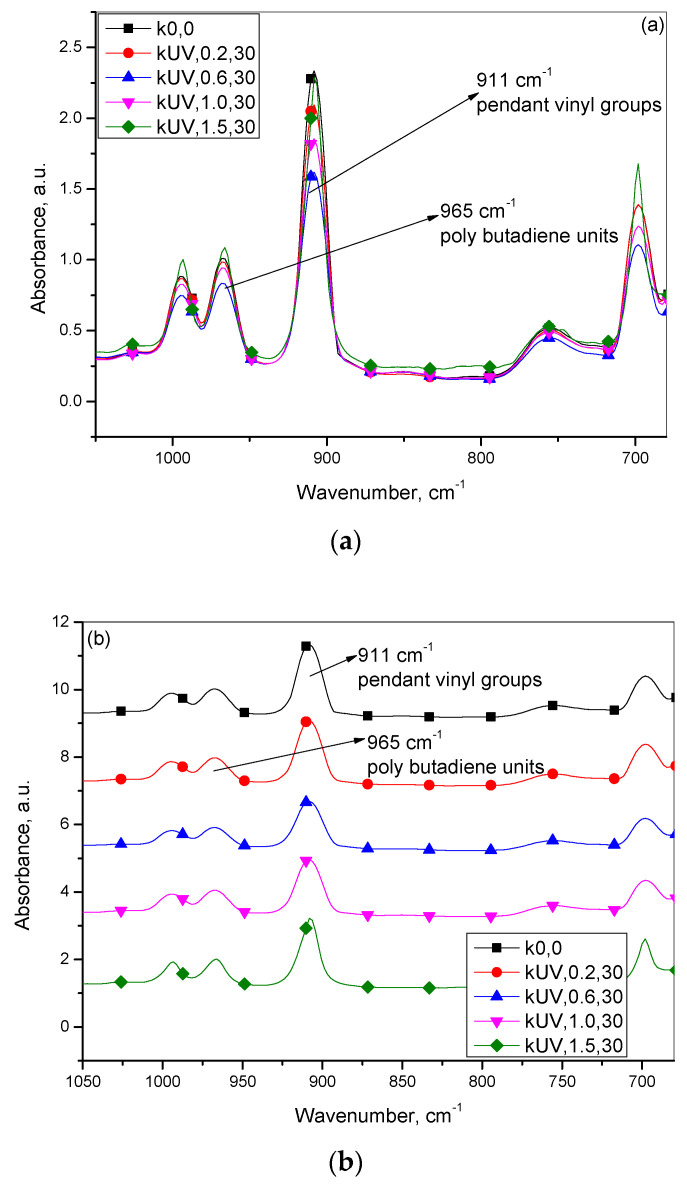
Un-subtracted spectra of the control sample and selected 30 s irradiated samples (**a**) superimposed and (**b**) the spectra stacked by Y offsets.

Furthermore, in order to support the assumption of crosslinking via vinyl double bonds in the UV-irradiated samples, ATR FT-IR studies were done. Figure 8a,b show the unsubtracted spectra of some selected samples in the wavenumber range of 1050 to 680 cm^−1^.

**Figure 9:**
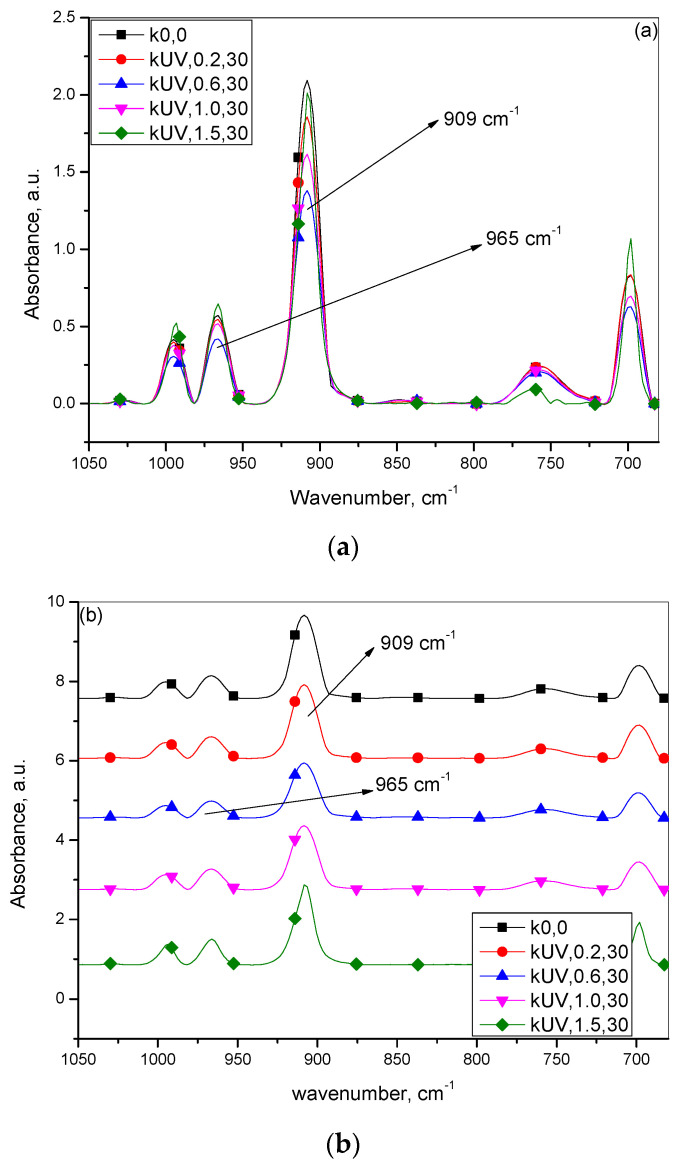
Baseline-fitted and subtracted spectra of the control sample and selected 30 s irradiated samples (**a**) superimposed and (**b**) the spectra stacked by Y offsets.

These spectra were of limited use in understanding the active participation of the dangling vinyl groups in photocrosslinking as they were not baseline subtracted. Thus, baseline subtraction was done by using an algorithm of baseline fitting and subsequent subtraction [20,21,22,23]. The baseline subtracted spectra of the same selected samples in the wavenumber range of 1050 and 680 cm^−1^ are presented in Figure 9a,b.

After baseline subtraction, the characteristic absorbance peak height for the main chain polybutadiene at 965 cm^−1^ was normalized and against this normalized peak height, the absorbance peak height of the vinyl pendant group at 909 cm^−1^ was calculated. The baseline subtracted peak heights and the subsequent calculations are presented in Table 4.

It is observed from the table that from the control sample to 1.0 phr of the photoinitiator, the ratio decreased in almost a linear manner. However, the ratio became almost the same for the 1.0 and the 1.5 phr photoinitiator compounded samples [15].

Since the ratio of the peak heights at 1 and 1.5 phr were almost the same, it was inferred that at this highest concentration, some unreacted photoinitiator remained on the surface of the polymer. The above analysis suggested that the vinyl double bonds were the active sites of crosslinking. In the process, some intramolecular cyclisation might have also taken place which is shown in Scheme 2.

**Figure 10:**
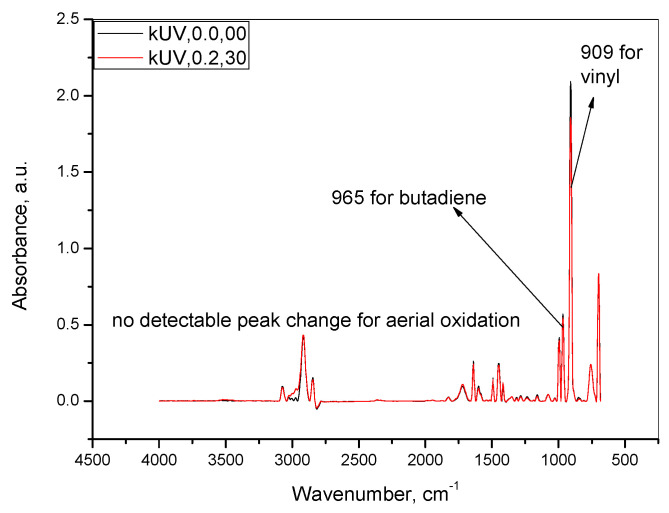
Superimposed, baseline subtracted spectra of the control sample and 0.2 phr incorporated 30 s irradiated sample to study oxidation of the UV-irradiated sample.

Superimposed, baseline subtracted FT-IR spectra of the control sample and the sample at photoinitiator concentration of 0.2 phr irradiated for 30 s, in the wavenumber range of 4500 to 650 cm^−1^ is shown in Figure 10. It depicts that there was no observable appearance of oxidized groups in the UV-treated sample. Thus, it proved that during the process of UV irradiation in air, no significant oxidation occurred.

These two baseline-subtracted spectra were already shown in Figure 9 amongst other spectra, but in a smaller wavenumber range

### 3.3. Surface Phenomenon through Contact Angle Studies

The disappearance of the vinyl pendant groups without any aerial oxidation was further supported through surface energy calculation (Table 5), by measuring equilibrium contact angle in selected solvents with the variously compounded and UV-crosslinked samples.

**Figure 11:**
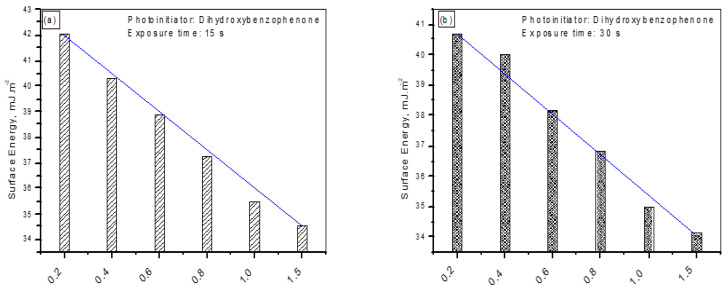
Surface energy as a function of photoinitiator concentration at (**a**) 15 s exposure time and (**b**) 30 s exposure time.

Since the maximum crosslinking occurred on the surface due to the phenomenon of cure depth gradient, contact angle measurements were done to calculate the surface energy to understand the changing nature of the surface. Figure 11a,b show surface energy as a function of photoinitiator concentration for the 15 s a nd 30 s crosslinked samples respectively.

It is seen from both figures that surface energy decreased with an increase in the photoinitiator concentration. Since $\gamma_{s(t)}$ which is the total surface energy of the solid sample is the sum total of $\gamma_{s}^{d}$ (dispersive part of the solid component) and $\gamma_{s}^{p}$(polar part of the solid component), a decrease in $\gamma_{s(t)}$ may be due to either a decrease in $\gamma_{s}^{d}$ or a decrease in $\gamma_{s}^{p}$ or a decrease in both. Table 6 shows that in the present case, the decrease in the total surface energy with an increase in photoinitiator concentration up to photoinitiator concentration of 1.0 phr was mainly due to a higher rate of decrease of the polar component.

In the polymer under study, polystyrene and the midblock 1,4 polybutadiene were non-polar while midblock 1,2 vinyl insertions contributed mainly to the polarity. This was due to the presence of sp^2^ hybridised carbon atom attached to sp^3^ hybridised carbon atom in the pendent vinyl groups. The faster rate of decrease of the polar component was then attributed to the disappearance of the pendent vinyl groups from the midblock polybutadiene during the process of crosslinking.

Also, with an increase in the photoinitiator concentration up to 1 phr, the total surface energy as well as the polar component, both decreased. Had there been residual photoinitiator, which was polar in nature due to the presence of carbonyl group and 4, 4′ hydroxy substitutions present on the surface of the polymer after crosslinking had taken place, then the polar component would have increased. This only happened at 1.5 phr.

This study conclusively revealed that with an increase in photoinitiator concentration up to 1 phr, more vinyl groups participated in photocrosslinking, with no residual photoinitiator remaining on the surface. A marginal increase in the polar component at 1.5 phr was attributed to some residual photoinitiator remaining unreacted at this concentration.

This unreacted mass was the reason behind failure through crack propagation. That is why the modulus value increased up to 1 phr and then decreased in all the cases.

Finally, it can be said that the polar component would not have decreased to such an extent or might have marginally increased, if, during the process of crosslinking, aerial oxidation would have occurred through which some carbonyl groups would have been formed. Thus, no such event markedly happened during the process.

### 3.4. SEM Studies on the Surfaces

**Figure 12:**
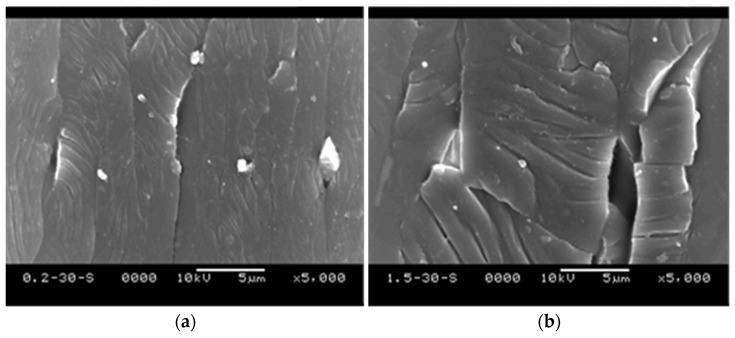
Surface photomicrographs of 15 s (**a**) and 30 s (**b**) UV-exposed polymer samples at 1.5 phr of photoinitiator concentration.

Surface analyses were performed by scanning electron microscopy to understand the surface changes due to irradiation. Figure 12a,b show the surface photomicrographs of 15 s and 30 s UV-exposed polymer samples respectively at 1.5 phr of photoinitiator concentration.

It was observed that the 15 s exposed sample showed the formation of some micro surface cracks which were much more pronounced in the case of the samples exposed for 30 s. From these observations it was inferred that along with the process of photoinitiator induced crosslinking which enhanced the tensile properties, photodegradation of the surface also occurred simultaneously under the condition of irradiation with UV light of given intensity and frequency. This resulted in the breakage of polymer bonds producing fragments [32]. Thus, the samples exposed to higher time, i.e., 30 s showed marginally lower tensile strength and modulus at equivalent photoinitiator concentrations when compared with the samples irradiated for 15 s.

## 4. Conclusions

The effects of ultraviolet radiation on the mechanical properties of a high vinyl SBS block copolymer were studied. The process variables were time of exposure to ultraviolet radiation and photoinitiator concentration in the polymer matrix at a fixed predetermined distance from the UV lamp.

The polymer showed positive reactivity towards ultraviolet radiation in the frequency range 250–350 nm in the presence of 4, 4′dihydroxybenzophenone as the photoinitiator. Both tensile strength and modulus showed improvement upon treatment with ultraviolet radiation over the control sample without any UV treatment. Even with an improvement over the control sample, the ultimate tensile strength decreased as a function of photoinitiator concentration while the modulus at 100, 200 and 300% increased from 0.2 to 1.0 phr of the photoinitiator concentration and then decreased at a concentration of 1.5 phr.

Inner shielding effect and some intramolecular cyclization were responsible for the reduction in the tensile properties. The overall balance of properties was thus a compromise between effective crosslinking and photoinduced degradation. The best results were obtained at a lower exposure time of 15 s and a photoinitiator concentration of 1 phr.

This study was purely experimental with an incorporated photoinitiator only, deliberately avoiding the use of any photosensitizer. This yielded instances of micrometer-thick crosslinking only. This very small thickness was effectively ascertained using a novel but very simple sol–gel experiment.

Further research aims in using a suitable photosensitizer along with the photoinitiator of interest.

## Figures and Tables

**Table 1:** Batch compositions in phr *.

	Sample Designation												
Components, phr *	k_0,0_	k_UV,0.2,15_	k_UV,0.4,15_	k_UV,0.6,15_	k_UV,0.8,15_	k_UV,1.0,15_	k_UV,1.5,15_	k_UV,0.2,30_	k_UV,0.4,30_	k_UV,0.6,30_	k_UV,0.8,30_	k_UV,1.0,30_	k_UV,1.5,30_
SBS block copolymer	100	100	100	100	100	100	100	100	100	100	100	100	100
Photoinitiator	0	0.2	0.4	0.6	0.8	1.0	1.5	0	0.2	0.4	0.6	0.8	1.0

* phr is parts per hundred rubber by mass.

**Table 2:** Literature data on contact angle probe liquid measurement.

Serial Number	Liquid	$\gamma_{d}^{l}\;(\mathrm{mN}\cdot m^{-1})$	$\gamma_{p}^{l}\;(\mathrm{mN}\cdot m^{-1})$	Reference
1	Formamide	39.5	18.7	26 Hefter (06)
2	Diiodomethane	48.5	2.3	27 Tang (2005)
3	Water	21.8	51.0	27 Tang (2005)

**Table 3:** Mechanical properties of the compounds at varying times and phoinitiator concentration irradiated with UV of a given frequency and intensity.

Components	k_0,0_	k_UV,0.2,15_	k_UV,0.4,15_	k_UV,0.6,15_	k_UV,0.8,15_	k_UV,1.0,15_	k_UV,1.5,15_	k_UV,0.2,30_	k_UV,0.4,30_	k_UV,0.6,30_	k_UV,0.8,30_	k_UV,1.0,30_	k_UV,1.5,30_
S-B-S	100	100	100	100	100	100	100	100	100	100	100	100	100
Photoinitiator	0	0.2	0.4	0.6	0.8	1.0	1.5	0.2	0.4	0.6	0.8	1.0	1.5
Mechanical Properties													
Hardness, Shore A	41	50	50	51	51	51	51	50	51	51	52	52	53
T.S. *, MPa	5.3	7.3	7.0	6.5	6.1	5.6	5.0	7.2	6.3	6.1	5.8	5.4	4.8
M ^#^ 100, MPa	0.7	0.9	1.0	1.2	1.3	1.3	1.0	0.7	0.8	1.0	1.2	1.3	0.9
M200, MPa	0.9	1.2	1.3	1.4	1.6	1.7	1.3	1.0	1.2	1.4	1.5	1.7	1.3
M300, MPa	1.2	1.6	1.7	1.8	2.2	2.2	1.7	1.3	1.6	1.8	2.2	2.2	1.7
E.B. ^$^, %	1200	1140	1090	1050	1030	1000	980	1130	1070	1010	1000	940	900
XLD ^&^, mol∙mL^−1^∙10^5^	­	2.37	2.90	3.51	3.95	4.39	4.86	2.41	2.92	3.67	3.89	4.43	4.87

* ultimate tensile strength; ^#^ modulus at a specified elongation % of 100, 200, 300; ^$^ elongation at break; ^&^ crosslink density.

**Table 4:** Baseline subtracted characteristic peak height ratios of vinyl to polybutadiene for the control sample and some selected for the 30 s UV-irradiated samples.

	Infrared Absorbance Peak Heights		
Sample Designation	Vinyl, 909 cm^−1^	Polubutadiene, 965 cm^−1^	Vinyl/Polybutadiene
k_0,0_	2.0944	0.5703	3.6728
k_UV,0.2,30_	1.8572	0.5451	3.4074
k_UV,0.6,30_	1.3804	0.4197	3.2889
k_UV,1.0,30_	1.6156	0.5178	3.1202
k_UV,1.5,30_	2.0113	0.6456	3.1155

**Table 5:** Surface energy (mJ·m^−2^) using water, formamide and diiodomethane as probe liquids.

Sample Designation	Water and Formamide	Formamide and Diiodomethane	Water and Diiodomethane
k_UV,0.2,15_	41.43	41.91	42.06
k_UV,0.4,15_	40.68	40.38	40.29
k_UV,0.6,15_	39.96	39.12	38.89
k_UV,0.8,15_	37.36	37.27	37.26
k_UV,1.0,15_	36.59	35.65	35.45
k_UV,1.5,15_	35.05	34.65	34.55
k_UV,0.2,30_	43.56	41.34	40.68
k_UV,0.4,30_	40.32	40.06	39.99
k_UV,0.6,30_	39.60	38.49	38.20
k_UV0.8,30_	35.89	36.64	36.82
k_UV,1.0,30_	36.59	35.29	35.00
k_UV,1.5,30_	35.05	34.30	34.11

**Table 6:** Dispersive and polar components of the total surface energy using water and diiodomethane as probe liquids.

Sample Designation	$\gamma_{t}\;(\mathrm{mJ}\cdot m^{-2})$	$\gamma_{d}\;(\mathrm{mJ}\cdot m^{-2})$	$\gamma_{p}\;(\mathrm{mJ}\cdot m^{-2})$
k_UV,0.2,15_	42.06	33.83	8.23
k_UV,0.4,15_	40.29	32.64	7.65
k_UV,0.6,15_	38.89	31.95	6.94
k_UV,0.8,15_	37.26	31.50	5.76
k_UV,1.0,15_	35.45	30.14	5.31
k_UV,1.5,15_	34.55	28.89	5.66
k_UV,0.2,30_	40.68	33.20	7.48
k_UV,0.4,30_	39.99	32.88	7.11
k_UV,0.6,30_	38.20	31.60	6.60
k_UV0.8,30_	36.82	30.89	5.93
k_UV,1.0,30_	35.00	29.51	5.49
k_UV,1.5,30_	34.11	28.26	5.85

## Data Availability

The data presented in this study are available on request from the corresponding author.
